# An analysis of errors in Chinese–Spanish sight translation by Chinese university students—a corpus study

**DOI:** 10.3389/fpsyg.2025.1516810

**Published:** 2025-10-07

**Authors:** Shiyang Liu

**Affiliations:** Foreign Language Faculty, China Foreign Affairs University, Beijing, China

**Keywords:** error analysis, Chinese–Spanish interpreting, sight translation, contrastive analysis, corpus-based interpreting research

## Abstract

Understanding the challenges inherent in Chinese–Spanish sight translation for undergraduate students is essential for enhancing their interpretation ability and accuracy. However, sight translation errors have rarely been studied, especially for Chinese–Spanish language pair. This study builds a corpus of Chinese university students’ Chinese–Spanish sight translation errors, which consists of 294 audio assignments and 2,923 error instances. The corpus of this study has three parameters: error levels, namely, the lexical, syntactic and grammatical; error manifestations that include substitution, addition, and omission; and source text analysis units, which are interpreting topics and sentences. Based on a combination of theories including the error analysis, Contrastive Analysis Hypothesis, the schema theory and Gile’s Effort Model, error analysis was conducted on the corpus to identify prevalent error types, analyze error distribution patterns, and determine error causes. This study employed a combination of quantitative and qualitative analysis. Frequency analysis, chi-square test and error rate were calculated to determine the prevalent error types and error distribution patterns. A qualitative analysis was also realized to determine the causes of errors. Results indicate that substitution was the most frequent error manifestation, whereas addition and omission were much less frequent. Regarding error levels, at the lexical level, sentences with difficult lexical expressions, like four-character words, abstract words, and poetic phrases, tend to concentrate more errors. Substitution of word selection and terminology, and omission of words were prevalent. At the syntactic level, sentence structure and omission of syntactic elements occurred most frequently, particularly in sentences with considerable length or complex structures. At the grammatical level, errors predominantly occur in areas where Chinese and Spanish have strong linguistic differences, such as agreement in gender and number, verb tense and conjugation, prepositions, and articles. The error causes constitute a complex mechanism that includes linguistic differences, negative translation of mother tongue, lack of domain knowledge and cultural understanding, cognitive load, and other factors. This study offers insights into error patterns and their causes in Chinese–Spanish sight translation, and provides a foundation for future studies on various areas in interpreting error analysis and interpreter training.

## 1 Introduction

Sight translation has a wide and diverse definition (Agrifoglio, 2004; Lambert, 1994; Pratt, 1991, quoted by Krapivkina, 2018, p. 696). Some linguists think that sight translation is the oral rendering of a text written in a source language (Dragsted and Hansen, 2009; Setton and Motta, 2007). Some consider it as a perfect exercise for interpreters and an important pedagogical tool in interpreting classrooms (Ho, 2022; Krapivkina, 2018; Lee, 2012; Li, 2014; Pöchhacker, 2004). The advantages of teaching sight translation to student interpreters include rapid source text analysis, avoidance of word-for-word translation, rapid conversion of messages from one cultural setting to another, improvement in note-reading and public-speaking skills, and enhanced flexibility of expression (Moser-Mercer, 1994; Weber, 1990). Sight translation is also effective in raising students’ awareness of syntactic and stylistic differences between source and target languages (Viaggio, 1995). In this research, in accordance with Jiménez Ivars and Hurtado Albir (2003), the concrete interpreting mode that we adopt is prepared sight translation, which permits interpreters to read the text before sight translation, and take notes about the vocabulary and expressions, and the syntactic order of the target text.

Considering the characteristics and the functions of sight translation, an analysis of errors is beneficial for identifying problems and weaknesses of student interpreters, analyzing the interpreting process, and understanding error patterns, which can contribute to reducing errors and improving interpreting accuracy. However, the error analysis of sight translation is rarely studied, especially for the language pair of Chinese and Spanish. One of the primary areas of focus in Chinese–Spanish interpreting error analysis is the categorization of errors that interpreters make during the interpreting process. Errors can manifest in various forms, including omissions, additions, substitutions, and distortions (Vargas-Urpí, 2018), and at various levels, like phonological level, lexical and clausal level, as well as syntactic and discoursal level (Zhao, 2019). The cognitive demands placed on interpreters in the Chinese–Spanish language pair should be particularly noteworthy, but there still lacks relevant studies for this language pair. Research has shown that cognitive load, language proficiency, and anxiety levels can significantly influence the occurrence of speech errors during consecutive interpreting sessions (Lozano-Argüelles et al., 2023). Furthermore, the language mastery is also important in effective interpreting. The structural differences between Chinese and Spanish can generate difficulties for interpreters and hence causing errors (Costa-jussà, 2015; Cao, 2020). The methodologies employed in studying interpreting errors generally incorporate qualitative and quantitative approaches (Vargas-Urpí, 2018; Costa-jussà et al., 2017) to gain a more comprehensive understanding of the error causes.

Considering the importance of sight translation error analysis in interpreting pedagogy and the insufficiency of studies for Chinese–Spanish language pair, this research intends to realize an error analysis of sight translation of Chinese university students specialized in Spanish, to analyze the error patterns and the error causes, with the purpose to improve interpreting accuracy.

## 2 Theoretical framework

This study employs a multi-theoretical approach to analyze sight translation errors. The Error Analysis theory provides the foundational methodology for error identification and classification, while the Contrastive Analysis, Schema Theory and Gile’s (1997) Effort Models are employed to analyze errors from three perspectives, the linguistic, the sociocultural and the cognitive. The Contrastive Analysis Hypothesis predicts and explains language-specific difficulties arising from structural differences between source and target languages. The Schema Theory, furthermore, reveals how interpreters’ knowledge structures influence their interpretations. The Gile’s Effort Models explains how cognitive resource allocation during simultaneous tasks may lead to errors when processing capacity is exceeded.

Error analysis has been considered a teaching resource in second language acquisition by many linguists (James, 1998; Corder, 1967, 1981; Fernández López, 1997; Lu, 1992; Santos Gargallo, 1993; Schachter, 1974; Sridhar, 1975; Vázquez, 1991, 1999). Regarding translation and interpreting training, error analysis is also of great value in discovering the interpreters’ weakness or insufficiency in linguistics ability as well as interpreting strategy, as it is believed that the symptoms of weakness or insufficiency, as well as translation mistakes, are also a precious pedagogical tool in the training of translators and interpreters (Gile, 1992; Nord, 1996). In the theoretical framework of error analysis, we should begin by discussing the definition and classification of errors. In the area of linguistic studies, abundant definitions of errors have been proposed from the perspective of second language acquisition (Corder, 1967; Dulay et al., 1982; James, 1998; Santos Rovira, 2007; Vázquez, 1999). However, in this section, we focus on reviewing the definitions of interpreting errors, as this is the focus of the present study. Barik (1975) believes that the interpreter’s version may depart from the original version in three general ways: omitting material uttered by the speaker, adding material to the text, or substituting material. Kopczynski (1981, quoted by Altman, 1994, p. 26) adopted the linguistic and communicative approaches simultaneously, proposing a definition that embraces both deviations from the linguistic norm of the target language and utterances that hamper the communication function of the speech act. Altman (1994) maintained that inaccuracies should be evaluated in terms of the extent to which they constitute an obstacle to communication, because the interpreter’s prime task is to communicate a message between the original speaker and a listener (or group of listeners) under a given set of circumstances. Falbo (1998) considers an error as a substitution of the cohesion and consistency of the source text in the interpreted text and of the equivalence in content and form between these two. Drawing on the aforementioned research, this study adopts the linguistic and communicative perspectives to define interpreting errors as deviations from the content of the original text and language norms of the target language, as well as hindrances to the communicative function of speech.

The Contrastive Analysis Hypothesis is a linguistic theory which was first proposed by Lado (1957) and compares the structures of two or more languages to identify their similarities and differences, predicting learning challenges according to the significant differences between the two languages. In interpreting studies, this approach is also applied to highlight the differences between source and target languages and provides insight into the prediction of interpreting challenges and the underlying causes of interpreting errors (Buansari et al., 2022; Khansir and Pakdel, 2019). Specifically, the Chinese and Spanish languages exhibit several differences in terms of morphology, vocabulary, grammar and syntax, which generates multiple interpreting challenges and errors. In this sense, the errors can be further explained through the lens of the negative transference theory, which posits that interpreters’ existing knowledge of their first language (L1) can interfere with their target language (L2) translation. This interference is particularly evident in sight translation, where interpreters must process written text in one language while simultaneously producing oral output in a second language. However, one limitation of this theory lies in its oversimplification of error causes to the linguistic differences. Given the multifaceted nature of the factors contributing to errors, contrastive analysis cannot solely attribute errors to linguistic differences (Ara, 2021; Spolsky, 1979). For this reason, we also employ the Schema theory to examine interpreters’ cognitive processes and their underlying knowledge, providing insight into how prior knowledge influences interpretation.

The Schema theory, presented by Piaget (1952), provides a valuable framework for understanding the cognitive processes involved in interpreting studies. This theory posits that individuals utilize pre-existing mental structures, or schemas, to organize and interpret new information. The interpretation process is inherently complex, involving various cognitive activities, such as comprehension, memorization, and expression, all of which are shaped by the interpreters’ existing schemas (Guo, 2022; Chen, 2024). Moreover, three types of schemata—linguistic, content, and formal, serve as the cognitive frameworks that influence how individuals interpret texts (Thang, 1997; Guo, 2022; Hu, 2018). Linguistic schemata are the phonetic, lexical, syntactic and semantic knowledge of the studied language. Content schemata describe the knowledge regarding the content area of the studied language. Formal schemata involve the form related aspects of the language. In the context of interpreting error analysis, errors often arise when individuals fail to activate the appropriate schemas or when their existing schemas are inadequate for interpreting new information. Moreover, research show that under stress, individuals are more likely to rely on already activated schemas, which can lead to errors if those schemas are not relevant to the task at hand (Vogel et al., 2017; Kluen et al., 2017). The Schema theory complements the Contrastive Analysis Hypothesis by examining the cognitive processes behind errors, explaining when and why errors occur due to a lack of mental schema integration, instead of only due to poor language transfer.

Gile’s Effort Models are developed to provide a theoretical framework for analyzing and understanding the cognitive processes and explain how cognitive overload during a language processing task can cause errors. According to Gile’s Effort Models, the sets of operations in interpreting process are grouped into “efforts,” which compete for a limited amount of processing capacity. In sight translation the interpreter is self-paced and faces less pressure on short-term memory with the source text visually present. Sight translation consists of “reading” a source-language text aloud in the target language, and Gile models it as “Sight Translation = Reading Effort + Memory Effort + Speech Production Effort + Coordination.” From this formula, we can see that in sight translation, listening and analysis effort simultaneously become a reading effort. The sight translator also faces the problem of segmenting the source text into meaning units and must fight against interference from the source language due to the absence of vocal indications and the presence of visual interference (Li, 2014). Regarding the memory effort involved in sight translation, although interpreters can control their rhythm of perception, smooth delivery is possible only when the interpreters begin reformulating while still reading. Syntactic differences between languages may force the interpreter to store information in memory until it can be appropriately inserted into the target language speech. This may pose little difficulty when the two languages are syntactically similar and when the source text is written in an easy-to-segment style, particularly when sentences are short and composed of independent clauses. When sentences are long and/or include embedded clauses, it may be necessary for the interpreter to read much more than one translation unit before reformulating it, which involves more time and effort in the reading component, as well as in short-term memory and coordination during production. This theory complements the error analysis and the Schema theory by pinpointing resource allocation issues within the cognitive processing of the interpreters, which contributes to finding the error causes.

## 3 Participants, materials and data analysis

### 3.1 Participants

The participants of the present study, 73 in total, were undergraduate students majoring in Spanish at the China Foreign Affairs University during their sixth and seventh semesters (aged between 21 and 22 years) when they received a one-year sight translation and consecutive interpretation training. The undergraduate students were Chinese, with Chinese as their native language and Spanish as their B language, mostly at Level C1 according to the Common European Framework of Reference for Languages (CEFR) levels, and English as their C language, mostly at Level B. Each student participating in this study was assigned a unique four-digit identification number. All errors in the corpus were marked with student IDs in the “ST” format, followed by four digits (e.g., ST1234), enabling the identification of error sources.

### 3.2 Materials

This study collected audio recordings of Chinese–Spanish sight translation sessions of the participants. The interpreting format employed in this study was sight translation. Before the translation, the students were given the interpreting theme to prepare, including to create vocabulary list, read background knowledge, etc. For the translation, students are provided with Chinese source texts on the spot and given 2 min to read through the material before rendering it into the target language. The preparation only permits making slashes for segmentation and marking important word meanings, and students cannot consult other resources or perform written translation. There were 19 original translation texts. Each text was translated by 9–20 students, and the entire corpus contained 294 audio assignments. To comprehensively analyze the difficulties of sight translation, we performed a quantitative analysis of the textual characteristics of the Chinese source texts. The HanLP and jieba were utilized for language processing, to calculate the length, tokenization (abbreviated as Token), semantic roles count (Sem. Rol), and dependency relation count (Dep. Rel). Four-character words (4CW) and terminologies (Trm) were tagged manually by the author, since online dictionaries do not incorporate the latest four-character phrases and neologisms. All statistics are shown in Table 1.

**Table 1:** Text properties of source texts.

Text code	Text topic	Sentence count	Character count	Token	4CW	Sem. Rol	Dep. Rel	Avg. sentence length	Avg. Token	Avg. Sem. Rol	Avg. Dep. Rel
TA	Artificial intelligence in Energy	6	298	169	3	80	169	49.67	28.17	13.33	28.17
TB	Rector speech	8	384	243	3	119	243	48.00	30.38	14.88	30.38
TC	Boao Forum	7	320	187	8	123	187	45.71	26.71	17.57	26.71
TD	China International Fair for Trade in Services	6	265	154	4	56	154	44.17	25.67	9.33	25.67
TE	The China-LAC Community with a shared future	7	354	203	7	105	203	50.57	29.00	15.00	29.00
TF	International Collaboration Forum	8	260	160	3	73	160	32.50	20.00	9.12	20.00
TG	National Bureau of Statistics	4	205	110	5	59	110	51.25	27.50	14.75	27.50
TH	New changes of studying abroad	7	481	299	12	137	299	68.71	42.71	19.57	42.71
TI	Chinese monetary policy	9	516	317	2	202	317	57.33	35.22	22.44	35.22
TJ	China International Import EXPO	9	263	152	3	86	152	29.22	16.89	9.56	16.89
TK	UN calls for emission reduction	9	381	210	6	149	210	42.33	23.33	16.56	23.33
TL	Studying abroad	10	361	216	19	116	216	36.10	21.60	11.60	21.60
TM	APEC Conference	8	318	192	4	120	192	39.75	24.00	15.00	24.00
TN	Concept of ¨One Belt One Road¨ Iniciative	6	309	190	0	137	190	51.50	31.67	22.83	31.67
TO	Energy conservation and environmental protection	6	262	148	4	68	148	43.67	24.67	11.33	24.67
TP	China-US relationship	8	347	220	1	126	220	43.38	27.50	15.75	27.50
TQ	China-EU collaboration	10	341	199	11	143	199	34.10	19.90	14.30	19.90
TR	China-Spain collaboration	7	326	188	5	103	188	46.57	26.86	14.71	26.86
TS	Green Industry in China International Fair for Trade in Services	6	252	155	2	78	155	42.00	25.83	13.00	25.83

Token, tokenization; 4CW, four-character word; Sem. Rol, semantic roles; Dep. Rel, dependency relationship; Avg. sentence length, average sentence length; Avg. Token, average tokenization; Avg. Sem. Rol, average semantic roles; Avg. Dep. Rel, average dependency relationship.

### 3.3 Data analysis

The author analyzed the collected audio recordings to evaluate errors in the students’ interpreting performance. This approach facilitates a contrastive analysis between the source and target texts, enabling an examination of students’ ability to transfer linguistic elements. In collating the error taxonomy, we reviewed existing error types, which include coherence, omission, addition (Barik, 1971; Deng, 2018; Falbo, 1998), lack of cohesion, clumsiness of expression (Falbo, 1998), logical errors, overelaboration, and self-correction (Deng, 2018). Other studies exist that aim to evaluate the interpreters’ abilities, such as proficiency in the second language and in the native language, knowledge of the topics being interpreted, ability to analyze and synthesize, mastery of interpreting strategies, communication skills (including active listening, clear expression, and empathy toward the speaker, relationships with the client and the audience, cultural adaptation, physical endurance, group work) (Deng, 2018; Novo Díaz, 2004). Based on previous studies and considering that the form of interpreting of the corpus is sight translation, and that the research objective is to evaluate the interpreter ability and language proficiency of students, this study employs the descriptive classification in the formulation of error taxonomy. The linguistic perspective serves as the primary framework for identifying and categorizing errors for that it offers a structured approach to classifying errors using concrete, measurable criteria. The errors are categorized into three main levels: lexical, grammatical and syntactic. Within each level, errors were further classified into three primary manifestations: substitution (the interpreter replaced part of the original text, causing the translation to deviate from the original content), omission (the interpreter omitted the content conveyed by the speaker), and addition (the interpreter added redundant content based on the original text). At each level, the sublevels were classified according to the pattern of interpreting errors detected in the corpus. At the lexical level, substitution errors encompass lexical use, selection, morphological inaccuracies, and terminological discrepancies. Syntactic errors relate to issues with omission and addition of syntactical elements, errors of sentence structure and word order, whereas grammatical errors include agreement in number and gender, verb tense and conjugation, subject-verb agreement, preposition, article, numerical errors and connector. The error taxonomy used in this study is presented in Table 2.

**Table 2:** Students’ sight translation error taxonomy.

Error level	Error manifestation	Sub-level (if applicable)	Explanation
Lexical	Omission		Omission of words that should have been translated from the original text.
	Addition		Addition of words that were not present in the original text.
	Substitution	Word selection	Semantic errors like incorrect word choice. Ambiguity.
		Terminology	Technical terminology. Chinese-specific terms. Personal names. Idiomatic expressions. etc.
		Word usage	Collocation errors and part-of-speech errors.
		Morphology	Neologism. Word-form distortion caused by memory or articulation errors.
Syntactic	Omission		Omission of a component. Clause. or entire sentence in the translation.
	Addition		Addition of components or clauses in the translation that were not present in the original text.
	Substitution	Sentence structure	Errors in sentence structure, such as mixing sentence patterns, improper use of sentence structures, incomplete sentences, and lack of logical coherence.
		Word order	Words, phrases, or clauses are not placed appropriately within the sentence, resulting in incorrect word order or phrasing.
Grammatical	Substitution Omission AdditionConnectors	Agreement in number and gender	Inconsistencies in the singular/plural form or gender (masculine/feminine) between nouns and their modifying adjectives.
		Verb tense and conjugation	Mistakes in verb tense, mood usage, or verb conjugation.
		Subject-verb agreement	Inconsistency between the subject and verb in terms of person.
		Prepositions	
		Articles	
		Numerals	
		Connectors	

Audio recordings were analyzed and the detected errors were categorized according to the established error taxonomy and transcribed into the corpus. Erroneous sentences were manually transcribed within the corpus, with the errors clearly marked. For each error in the corpus, the following parameters were marked: student ID, article topic, sentence number, sentence containing the error, error manifestation, error level, and error sub-level if applicable. The author systematically identified and categorized interpreting errors based on the established error taxonomy. Subsequently, a corpus of students’ Chinese–Spanish interpretation errors was compiled, totaling 2,923 instances. Statistical analysis was conducted using python to examine the distribution of errors across various error levels, concrete error types, error manifestations, and sentence numbers in each original text. A qualitative analysis was also realized based on the theoretical framework to understand the error causes and characteristics. Therefore, the error analysis based on this taxonomy significantly advances the field by identifying common challenges and interpreter weaknesses, analyzing cognitive processes, and enhancing quality control criteria. It addresses a critical gap in existing taxonomies (Falbo, 1998; Kopczynski, 1981; Gile, 1987), which have overlooked the specific errors encountered in Chinese–Spanish sight translation.

## 4 Quantitative analysis results

This corpus that we established accounts for four parameters: error manifestation, error level, interpreting topic and sentence number. The first two parameters define the error taxonomy, structuring the quantitative analysis and the discussion; the last two are parameters of the source text, which serve as unit of error analysis. In the quantitative analysis, this study performed a cross-tabulation analysis and frequency analysis with python to analyze the distribution pattern of errors and the correlation between different error parameters. The error rate (ER) was also calculated to detect the distribution pattern of errors in different texts and sentences, as well as at different error levels and error manifestations in the same text, considering the differences in the number of participants and text length (including the count of Chinese characters, numerals, spaces and punctuation) of the source texts. The equation used to calculate the error rate is:


$$\text{standard error rate}=\frac{\text{number of errors}}{\text{text length}\times \text{number of participants}}\times 100\%$$

### 4.1 Error level

It is important to note that although errors at lexical, syntactic and grammatical levels show different proportional distributions in the corpus, no direct comparisons of error prominence across these levels or inferences about students’ translation difficulties can be made due to the varying total occurrences of words, sentences, and grammatical structures in the corpus. In this study, error analysis can only be conducted by comparing prominent error sublevels within each respective level. As shown in Table 3, the most frequently committed errors for each level were as follows: At the lexical level, word selection (670 errors, 37.85% of lexical errors) was the most frequent error. Substitution of terminology (19.32%) and word omission (18.64%) were other frequent problems. At the syntactic level, the predominant errors observed were substitutions related to sentence structure (237 errors, 65.29% of sentence errors) and omissions of syntactic elements (106 errors, 31.27%). The grammatical errors made by students are predominantly concentrated in agreement in gender and number (280 errors, 35.44%), verb tenses and conjugation (212 errors, 26.84%), prepositions (88 errors, 11.14%), and articles (68 errors, 8.60%). Regarding the error manifestation, the three levels show a similar error distribution pattern: high percentage of errors in substitution, followed by omission, and least amount of addition errors. Only at the syntactic level, omission errors are relatively more (30.02% of all syntactic errors), compared with the other two levels.

**Table 3:** Distribution of errors in error sublevels.

Error level	Error manifestation	Sub-level (if applicable)	Error count	Percentage of error level
Lexical	Substitution	Word selection	670	37.85%
		Terminology	342	19.32%
		Word usage	231	13.05%
		Morphology	162	9.15%
	Omission		330	18.64%
	Addition		35	1.98%
Syntactic	Substitution	Sentence structure	237	65.29%
		Word order	15	4.13%
	Omission		109	30.03%
	Addition		2	0.55%
Grammatical	Substitution	Agreement in number and gender	280	35.44%
	Substitution	Verb tense and conjugation	212	26.84%
	Substitution	Subject-verb agreement	97	12.28%
	Substitution	Prepositions	55	6.96%
	Omission		27	3.42%
	Addition		6	0.76%
	Omission	Articles	26	3.29%
	Addition		24	3.04%
	Substitution		18	2.28%
	Substitution	Numerals	31	3.92%
	Substitution	Connectors	13	1.65%
	Addition		1	0.13%

Regarding the correlation between interpreting topics and the error level, with a Pearson Chi-square value of 2.46% (df = 36) and a *p*-Value of 1.0, we find that the error rate of the lexical, syntactic and addition variables across different topics do not show a significant difference. This suggests that the topical domain is not a decisive factor in determining error patterns across various linguistic levels. A more detailed examination at the sentence level is required to understand the underlying causes of these interpretation errors. Regarding the 141 sentences that constitute our entire corpus, the median error rates of the lexical, syntactic and grammatical levels are, 1.83, 0.40, and 0.81%, respectively, while the highest error rates are 5.88, 2.22, and 3.70%, respectively. The median error rates across all three levels are below 2%, indicating a relatively low overall percentage. However, the highest error rates of sentence, ranging from 2.22 to 5.88%, indicate a significant increase in errors in individual cases. Moreover, both the median and maximum values of ER at the lexical level are notably higher than the other two levels, which, however, does not necessarily indicate that lexical errors are more significant than those at other levels, as the frequency of lexical items in a single sentence typically exceeds the number of syntactic structures and grammatical occurrences.

### 4.2 Error manifestation

According to the frequency analysis, as shown in Table 4, based on different error manifestations, there were 2,363 cases of substitution errors in the corpus, accounting for 80.84% of the total errors. Omission and addition errors were relatively fewer, with 492 (16.83%) and 68 errors (2.33%), respectively. This implies that the primary issue for students in interpreting is their inability to translate the original text accurately with the correct expressions, with fewer instances of problems with redundancy or omission. The error distribution pattern presented different tendencies. In substitution, lexical errors occupied the main part (59.46%), followed by grammatical (29.88%) and syntactic errors (10.66%). In omission, lexical errors were the main part (67.07%), followed by syntactic errors (22.15%) and grammatical errors (10.77%). Regarding addition errors, the percentage of lexical and grammatical errors are relatively close, at 51.47 and 45.59%, respectively.

**Table 4:** Distribution of errors in different error levels and error manifestations.

Category	Substitution	Omission	Addition	Total
Lexical	1,405 (79.38% within Lexical) (59.46% of Substitution)	330 (18.64% within Lexical) (67.07% of Omission)	35 (1.98% within Lexical) (51.47% of Addition)	1,770 (100% within Lexical) (59.77% of Total)
Grammar	706 (89.37% within Grammar) (29.88% of Substitution)	53 (6.7% within Grammar) (10.77% of Omission)	31 (3.92% within Grammar) (45.59% of Addition)	790 (100% within Grammar) (26.51% of Total)
Syntactic	252 (69.42% within Syntactic) (10.66% of Substitution)	109 (30.02% within Syntactic) (22.15% of Omission)	2 (0.55% within Syntactic) (2.94% of Addition)	363 (100% within Syntactic) (11.60% of Total)
Total	2,363 (80.84% of Total) (100% of Substitution)	492 (16.83% of Total) (100% of Omission)	68 (2.33% of Total) (100% of Addition)	2,923 (100% of Total)

The Chi-square test was conducted to examine the association between interpreting topics and error manifestation. With a Pearson Chi-square value of 2.34% (df = 36) and a *p*-Value of 1.0. We find that the error rate of the substitution, omission and addition variables across different topics do not show a significant difference. This result is consistent with the correlation findings among error levels, suggesting that the subject matter of interpretation is not a major determinant of students’ error patterns across different error manifestations. Regarding different sentences, the median error rate of substitution, omission and addition levels are 2.46, 0.52, and 0.07%, respectively, while the highest error rates are 6.72, 2.43, and 0.79%, respectively.

### 4.3 Text analysis

The error rate of the 19 original texts was also calculated to discover the patterns of texts, as shown in Table 5. Comparing the text properties in Table 1 and the error rates in Table 5, no correlations were found, implying that the concentration of errors does not have a clear correlation with the translation topics. Error rates are influenced more by individual sentence structures and expressions rather than by specific textual characteristics. Therefore, the discussion section will provide a qualitative analysis of the sentences that concentrate the highest error rate.

**Table 5:** Error count and error rate of error levels and error manifestations of each text.

Text code	Sub.	Add.	Omi.	Lex.	Synt.	Gram.	Total errors	Participant	ER of text
TA	116	0	14	97	17	16	130	18	2.42%
	2.16%	0.00%	0.26%	1.81%	0.32%	0.30%			
TB	113	5	26	72	31	41	144	14	2.71%
	2.12%	0.09%	0.49%	1.35%	0.58%	0.77%			
TC	180	8	55	152	23	68	243	18	4.29%
	3.17%	0.14%	0.97%	2.68%	0.41%	1.20%			
TD	188	4	60	169	23	60	252	17	5.88%
	4.39%	0.09%	1.40%	3.94%	0.54%	1.40%			
TE	57	1	20	46	12	20	78	16	1.04%
	0.76%	0.01%	0.27%	0.62%	0.16%	0.27%			
TF	97	4	15	55	23	38	116	16	2.77%
	2.31%	0.10%	0.36%	1.31%	0.55%	0.91%			
TG	129	6	16	96	8	47	151	17	4.42%
	3.78%	0.18%	0.47%	2.81%	0.23%	1.38%			
TH	48	1	11	36	4	20	60	9	1.37%
	1.10%	0.02%	0.25%	0.82%	0.09%	0.46%			
TI	187	7	52	162	27	57	246	18	2.61%
	1.98%	0.07%	0.55%	1.72%	0.29%	0.60%			
TJ	55	0	7	42	6	14	62	16	1.47%
	1.30%	0.00%	0.17%	0.99%	0.14%	0.33%			
TK	67	0	2	38	9	22	69	12	1.61%
	1.56%	0.00%	0.05%	0.89%	0.21%	0.51%			
TL	99	0	21	69	25	26	120	15	2.22%
	1.83%	0.00%	0.39%	1.27%	0.46%	0.48%			
TM	100	1	18	81	16	22	119	13	2.92%
	2.46%	0.02%	0.44%	1.99%	0.39%	0.54%			
TN	169	0	15	109	37	38	184	14	4.17%
	3.83%	0.00%	0.34%	2.47%	0.84%	0.86%			
TO	110	3	41	81	20	53	154	15	4.01%
	2.86%	0.08%	1.07%	2.11%	0.52%	1.38%			
TP	120	2	38	88	24	48	160	17	2.81%
	2.11%	0.04%	0.67%	1.55%	0.42%	0.84%			
TQ	222	13	29	146	24	94	264	18	4.30%
	3.62%	0.21%	0.47%	2.38%	0.39%	1.53%			
TR	218	12	42	163	22	87	272	17	4.97%
	3.98%	0.22%	0.77%	2.98%	0.40%	1.59%			
TS	88	1	10	68	12	19	99	14	2.81%
	2.49%	0.03%	0.28%	1.93%	0.34%	0.54%			

Sub., substitution; Add., addition; Omi., omission; Lex., lexical; Synt., syntactic; Gram., grammatical.

## 5 Discussion

In this section, we will discuss the results of the quantitative analysis and further examine a selection of sentences qualitatively. This qualitative analysis will explore the underlying error patterns and causes by focusing on the 10% of sentences with the highest error rate for each error level, as well as the most prominent error sub-levels.

### 5.1 Error level

As shown in the Table 6, sentences with high lexical error rate (LER) do not demonstrate notable values in their textual parameters. It is particularly noteworthy that most of these sentences do not contain a large number of four-character idioms or technical terms. Only the TD_5,^1^ which lists technical terms, contains six specialized terms and new words. Our qualitative analysis shows that the sentences with high lexical error rates have the following characteristics: First, sentences with high LER may contain abstract words. Borghi and Binkofski (2014) define abstract words as those that denote intangible and imperceptible objects and concepts grounded in physical entities and concrete, singular objects. Due to their complexity (Barsalou, 2003) and semantic variability, students often struggle to precisely understand their meanings and to find appropriate Spanish equivalents. Sentences that exhibited errors of this type include TR_5, TM_2, TD_4, TS_6, and TQ_6. For example, in TR_5, students encountered difficulties in finding the Spanish equivalent of “相关单位” [entidades pertinentes]^2^ and either omitted entire words or parts of them (ST1756, 1761, 1748). Other students made errors by choosing words with similar forms or by confusing synonyms, as in the case of “unidades relevantes” (相关单位) (ST1748, 1749), “unidades relativas” (相对单位) (ST1759), “autoridades pertinentes” (有关当局) (ST1758), “institutos competentes” (主管机构) (ST1743). Another typical example of error of this cause is the interpretation of “新愿景” [la nueva visión] in TM_4. This expression emphasizes a new perspective and expectation, rather than just a hope. For this reason, “nueva esperanza” (新希望) (ST1753, TM_4) cannot translate the meaning of guiding vision or strategic plan. Likely, “nueva previsión” (新预测) (ST1747, TM_2) refers to a forecast, prediction, or provision, which implies a more specific and concrete estimation of future events or needs. Such errors, coming from abstract words, show that students struggle to accurately understand the meanings of words in both the source and target languages within specific contexts (Xiang and Zheng, 2018). Different semantic fields between languages cause confusion in synonyms selection, increasing the coordination load for interpreters. The limited linguistic and cultural schema of the students also hindered their ability to find corresponding translations for the original text.

**Table 6:** The 10% of sentences with the highest lexical error rates and their textual characteristics^1^.

Sent ID	Source text sentence	Sent Len	Token	4CW	Trm	Sem. Rol	Dep. Rel	LER
TR_5	向对本次活动给予大力支持的*相关单位*和参会企业表示*衷心的感谢*! [También me gustaría agradecer a las instituciones concernientes y las empresas participantes que han brindado un fuerte apoyo a este evento.]	30	19	0	0	18	19	5.88%
TD_5	*数字贸易, 5G通讯, 工业互联网, 智慧办剬, 区块链创新, 跨境电商*…… [Comercio digital, comunicaciones 5G, Internet industrial, oficina inteligente, innovación blockchain, comercio electrónico transfronterizo …]	31	21	0	6	0	21	5.50%
TM_4	比如, 根据*新愿景*, 未来APEC将推动数字经济*新业态*新模式发展。[Por ejemplo, de acuerdo con la nueva visión, APEC promoverá el desarrollo de nuevos formatos y modelos de la economía digital en el futuro.]	31	18	0	2	7	18	4.96%
TD_4	在今年的服贸会上, 覆盖服务贸易全部12大*领域*的展览, 展示了中国服务贸易行业的繁荣*全貌*。[En la Feria de Servicios de este año, las exhibiciones que cubren los 12 campos del comercio de servicios mostraron la prosperidad panorámica de la industria del comercio de servicios de China.]	43	26	0	2	9	26	4.79%
TG_3	“总的来看, 10月份国民经济持续恢复增长, *‘六稳”六保’*任务落实成效进一步显现。” [“En general, la economía nacional continuó reanudando su crecimiento en octubre, y la implementación de las tareas de ‘seis estabilidad’ y ‘seis garantías’ ha demostrado aún más su efectividad.”]	41	22	2	1	10	22	4.73%
TF_2	*金秋*九月, *丹桂*飘香。 [En el septiembre del otoño dorado, estamos rodeados por el osmanthus fragante.]	10	6	1	0	3	6	4.38%
TO_5	国家节能中心*副主任*杨博表示, 近年来中国节能环保产业*增加值*以*年均15%左右*的速度增长。 [Yang Bo, subdirector del Centro Nacional de Ahorro Energético, dijo que en los últimos años, el valor agregado de la industria de ahorro energético y protección ambiental de China ha aumentado a una tasa anual promedio de alrededor de 15%.]	42	22	1	1	7	22	4.29%
TN_2	*开放包容, 合作共赢*是*“一带一路”*倡议的核心理念。 [La apertura, la inclusión y la cooperación de beneficio mutuo son los conceptos nucleares de la iniciativa la Franja y la Ruta.]	24	16	3	0	3	16	4.17%
TN_1	我们期待, 以*开放包容*, 合作共赢理念为引领, 推动*构建*更加剬正, 合理和均衡的全球治理体系。 [Esperamos construir un sistema de gobernanza global más justo, razonable y equilibrado guiado por el concepto de apertura, inclusión y cooperación de beneficio mutuo.]	43	26	2	1	19	26	4.15%
TD_3	据统计, 本届服贸会吸引了包括近200家*世界500强企业*在内, 来自148个国家和地区的2.2万家企业和机构*线上线下*参展参会。 [Según las estadísticas, esta feria de servicios ha atraído a 22.000 empresas e instituciones de 148 países y regiones, incluidas casi 200 empresas de Fortune 500, para participar en línea y fuera de línea a esta exposición.]	61	35	0	2	11	35	4.15%
TD_6	服贸会上的*新技术, 新业态*吸引着外媒的关注。 [Las nuevas tecnologías y nuevos formatos en la Feria de Servicios han atraído la atención de los medios extranjeros.]	21	14	1	1	5	14	3.92%
TG_1	11月16日, 国家统计局发布了10月份国民经济运行情况。 [El 16 de noviembre, la Oficina Nacional de Estadísticas publicó la situación de operación económica nacional de octubre.]	28	15	1	1	6	15	3.78%
TS_6	绿色发展新动力不断*释放*, *实实在在*的绿色服务效益正不断惠及民众。 [El nuevo ímpetu para el desarrollo verde se libera constantemente y los beneficios tangibles de los servicios verdes benefician continuamente a las personas.]	31	17	1	1	8	17	3.69%
TQ_6	随着新冠疫情不断*蔓延*, 全球*经济社会运转*遭到严重冲击, 引发*复杂, 动荡*的局面。 [A medida que la nueva epidemia de la corona continúa ampliándose, las operaciones económicas y las sociedades mundiales se han visto gravemente afectadas, provocando una situación compleja y turbulenta.]	37	21	2		13	21	3.45%

1 Based on common errors committed by students in the corpus, the author highlights the challenging parts in each sentence in italics, corresponding to the error level or error manifestation in the table. Accompanied by the metrics of text properties on the right side of each sentence, it helps readers understand the potential sources for these errors.

Sent ID, text code_sequence number of the sentence in the text; Sent Len, sentence length; Token, tokenization; 4CW, four character word; Trm, terminology; Sem. Rol, semantic role; Dep. Rel, dependency relationship; LER, lexical error rate.

Second, errors tend to occur in sentences exhibiting a high density of professional terminology, neologisms, or specialized expressions within a particular domain. Prior to the translation task, participants were informed of the subject matter and could conduct pre-translation preparation, including terminology studying. However, they were not aware of the specific terms and proper nouns that would appear in the source texts. Therefore, the mastery of terminology is, to some extent, closely related to the thoroughness of interpreters’ preparation and the comprehensiveness of their established terminology database.

Table 6 shows that the terminologies of new technology and neologisms represent a challenge to the vocabulary and background knowledge of the interpreters. For instance, in TD_5, errors are detected with the relevant terminologies showcased in China International Fair for Trade in Services. “数字贸易” [comercio digital] is erroneously interpreted as “comercio de inteligente” (聪明贸易) (ST1747), “transacción digital” (数字交易) (ST1757) or “comercios internet” (互联网贸易) (ST1743). “5G通讯” [comunicaciones 5G] registers an error as “comunicación según G” (根据G通讯) (ST1747). “智慧办剬” [oficina inteligente] has various interpretations, such as “oficinas intelectuales” (智力办剬室) (ST1756), “oficio inteligente” (智慧型职业) (ST1761), “trabajo de inteligencia” (智能劳动) (ST1733), “trabajo artificial” (人工劳动) (ST1743), and “gestión inteligente” (智慧管理) (ST1757). While some of these interpretations convey the general idea, they do not accurately capture the concept of “intelligent office.” “跨境电商” [comercio electrónico transfronterizo] is interpreted erroneously as “comercios electrónicos internacionales” (国际电子商务) (ST1756), “comercio online de transporte” (运输服务在线交易) (ST1749), etc. In summary, the main issues with the interpretations of these technical terms and neologisms are inconsistency, inaccuracy, and the use of nonstandard or improvised interpretations that do not effectively convey the original Chinese concepts.

In sentences of TD_6 and TM_4, the Chinese neologism “新业态” [nuevos formatos] and “新模式” [nuevos modelos] create difficulty for interpreters to understand precisely the meaning and find the suitable Spanish words. Errors are detected such as “la nueva normalidad y modalidad” (新常态与新模式) (ST1755), “nuevos formatos y modos” (新形式与新方式) (ST1754), “una nueva situación y un nuevo modelo” (新形势与新模型) (ST1750), “nuevo tipo de negocio” (新型业务) (ST1733), “nuevos aspectos” (新方面) (ST1745), and “nueva moda y nueva industria” (新时尚与新产业) (ST1747).

Moreover, the substitution of professional terminology, including institutional names and economic terminology detected in our corpus, significantly contributes to translation errors due to students’ unfamiliarity with Spanish expressions and their lack of corresponding linguistic-cultural knowledge. For example:

博鳌亚洲论坛同澳门特别行政区政府共同举办国际科技与创新论坛大会, 将为全球科技创新提供交流合作的重要平台。(TC_6, SenT. Len 52, 4CW 0, Trm 3, Sem. Rol 9, Dep. Rel 29, LER 2.35%)

[El Foro de Boao para Asia y el Gobierno de la Región Administrativa Especial de Macao albergan conjuntamente el Foro Internacional sobre Tecnología e Innovación, que proporcionará una plataforma importante para los intercambios y la cooperación en la innovación científica y tecnológica mundial.]

In the sentence TC_6, “澳门特别行政区” [el Gobierno de la Región Administrativa Especial de Macao] is interpreted as “distrito Aomen” (澳门区) (ST1743), “región especial Aomen” (澳门特区) (ST1746) or “la comunidad especial de Aomen” (澳门特别社区) (ST1753).

In TG_4, “国家统计局” should be translated as “*Instituto Nacional* de Estadísticas” or “Oficina Nacional de Estadísticas,” but students did not comprehend the expression and invented words:

国家统计局新闻发言人付凌晖表示, 要按照党中央, 国务院的决策部署, 进一步统筹好新冠肺炎疫情防控和经济社会发展, 促进经济持续稳定复苏, 为“十四五”开局奠定良好基础。(TG_4, SenT. Len 81, 4CW 2, Trm 6, Sem. Rol 23, Dep. Rel 44, LER 1.60%)

[Fu Linghui, portavoz de la Oficina Nacional de Estadísticas, dijo que de acuerdo con la decisión y el despliegue del Comité Central del Partido y el Consejo de Estado, coordinarán aún más la prevención y el control de la nueva epidemia de la corona virus y el desarrollo económico-social, promover la recuperación económica sostenida y estable y sentar una buena base para el inicio del “XIV Plan Quinquenal”.]

Errors were detected such as “Ministerio de Estadísticas” (统计部) (ST1762), “Oficina Nacional de Estrategia” (国家战略办剬室) (ST1754) and “Centro Nacional de Estadísticas” (国家统计中心) (ST1758). In TO_5, there are relevant cases of errors of the economic terminology “增加值” [valor añadido], which is translated as “el valor acelerado” (加速值) (ST1763), “el volume total de producción” (总产量) (ST1761), or just omitted (ST1751, 1756). The main causes of terminology errors include a lack of terminological and cultural schemata both in Chinese and Spanish. This unfamiliarity and insufficiency also increase the cognitive load for the interpreters, leading to omissions or inaccuracies in their translations (Hardy et al., 2013; Horváth, 2019). These findings agree with the equivalent recall discussed by Putranti (2017).

The third type of sentence involves common sayings, *chengyu* fixed expressions, and poetic phrases. In TF_2, errors arise from the inability to transfer semantic relations, such as interpreting “金秋九月” [septiembre dorado de otoño] as “En el otoño dorado y el septiembre,” (在金色的秋天和九月) (ST1756), and “en este otoño de oro” (在这个金色的秋天) (ST1755). Other students fail to select the correct Spanish word to translate “丹桂” [osmanthus fragante], interpreting it as “flores” (花) and “árboles” (树) (ST1755, 1743).

Furthermore, certain fixed formulations or collocations exhibited a four-character pattern, predominantly within the political domain. The compact structure, its incisive meaning, and the concentrated embodiment of linguistic rhetoric and cultural accumulation (Kang and Yang, 2022) are characteristics of these idioms, and cause interpreting difficulties for students. For example, in TG_3, “六稳” “六保” [las “seis estabilidades” y las “seis garantías”] is a numeric abbreviation which is often used in Chinese political texts. Its concise expression and cultural and political context create a high level of difficulty for understanding and translating. Error causes can include unfamiliarity with the background knowledge and the significance of the term, and committing errors, such as “seis estabilidad y seis seguridad” (六稳六保) (ST1733), “seis estabilizaciones y seis garantías” (六稳六保) (ST1753), and “seis estabilidad y las garantías” (六稳和保障) (ST1762). In TN_2, the interpretation of “开放包容” [apertura e inclusión] led to errors like wrong selection and formation of the words “la apertura e inclugencia” (开放与包容) (ST1478), “la tolerancia abierta” (开放的包容) (ST1464), “la abirdad y tolerancia” (开放和宽容) (ST1479), and “la actitud tolerante y abierta” (宽容开放的态度) (ST1476), as well as omission of information – “la abierta” (开放) (ST1469).

Apart from the typical sentences that contain higher LER, word selection, substitution of terminology and omission of words are the three lexical sublevels with prominent errors (as shown in Table 4). The reason for the concentration of errors in word selection may be insufficient vocabulary comprehension (Jiménez Ivars and Hurtado Albir, 2008), and multiple similar vocabulary choices may exist, as discussed in the part of abstract words. Interference by the translator’s mother tongue (intralingual interference) and lack of linguistic schemata of the target language can also cause translation errors (Phạm, 2018; Utami, 2017). Moreover, words with abstract meanings and common sayings are more difficult to process cognitively than concrete words. Research has shown that concrete words are more easily remembered and processed than abstract words, which may contribute to higher error rates in interpreting abstract concepts (Fliessbach et al., 2006). The terminology error has been analyzed in the second type of high LER, while the third sublevel, omission, is spread across different sentences, mainly due to lack of understanding of the correct use of the Spanish expressions, or failing to remember the expression and omitting information, as is shown in TC_5:

中国愿同各国一道, 加强科技创新与合作, 促进更加开放包容, 互惠共享的国际科技创新交流, 为推动全球经济复苏, 保障人民身体健康作出贡献。(TC_5, SenT. Len 65, 4CW 2, Trm 0, Sem. Rol 39, Dep. Rel 39, LER 3.16%)

[China está dispuesta a trabajar con otros países para fortalecer la innovación y la cooperación tecnológica, promover intercambios internacionales de innovación tecnológica más abiertos, inclusivos y mutuamente beneficiosos, y contribuir a promover la recuperación económica mundial y garantizar la salud del pueblo.]

In this sentence words like “创新” [innovación] (ST1761), “合作” [cooperación] (ST1733), “包容” [inclusión] (ST1749, 1747, 1753), “复苏, 保障” [recuperar, garantizar] (ST1753) and “人民” [el pueblo] (ST1762) are omitted. Word usage errors are also detected, including translating “愿” [estar dispuesto a] as “tiene muchas ganas para” (非常渴望) (ST1743), and “为…保障人民身体健康作出贡献” [contribuir a la salud del pueblo] as “contribuir (a^*^^3^) la protección de la salud de los ciudadanos” (为保护剬民健康做出贡献) (ST1743).

At the syntactic level, similarly, we detected the 10% sentences with the highest syntactic error rate (SYER), as shown in Table 7. Firstly, the sentences including poems or ancient sayings present a higher level of difficulty for interprets’ understanding and skills, therefore leading to more errors. The sentences TB_4, TF_2, and TE_4 are relatively low in sentence length, semantic roles, and dependency relationship, but their condensed and context-dependent expressions, culture-specific allusions and references, as well as the parallel structure signify difficulties for interpreters. In TB_4, the main detected error is omission, meaning that interpreters tend to omit part of the sentence for evasion due to the difficulty of understanding the Spanish expression in Chinese. Incomplete sentences were found like “cincuenta años de viento y lluvia inusuales, cincuenta años de arduo trabajo.” (五十年风雨不寻常, 五十年艰苦奋斗) (ST1745), “durante el trabajo de más de cincuenta años” (在五十多年的工作中) (ST1733), “han transcurrido cincuenta años de singularidad y de cultivo duro” (经历五十年的非凡与艰辛耕耘) (ST1761), “tras cincuenta años de trabajo duro” (五十年的艰苦奋斗后) (ST1762), and “cincuenta años de aventura y esfuerzos son inusuales” (五十年的冒险与努力是非凡的) (ST1749). In TF_2, the errors include “En el septiembre con la aroma de la flora” (在充满花香的九月) (ST1761), “En el otoño de septiembre” (在九月的秋天) (ST1749), and omission of the whole sentence (ST1754). In TE_4, we detected omission of the whole sentence (ST1856), as well as the following substitution errors, indicating sentence structure distortion and ambiguous expressions in Spanish, like “Amigos con ambiciones no se llevan las montañas y los mares tan lejos.” (志同道合的朋友不嫌山海远) (ST1862), and “Incluso los voluntario no para montañas y mares lejos.” (连志愿者也不因山海遥远而却步) (ST1845).

**Table 7:** The 10% of sentences with the highest syntactic error rates and their textual characteristics.

Sent ID	Source text sentence	Sent Len	Token	4CW	Trm	Sem. Rol	Dep. Rel	SYER
TL_2	又有哪些新的变化? [¿Cuáles son los nuevos cambios?]	9	7	0	0	7	7	2.22%
TB_4	五十年*栉风沐雨不寻常*, 五十年*辛勤耕耘谱华章*。 [Cincuenta años de arduo trabajo son inusuales, y componen un capítulo brillante.]	22	13	2	0	11	13	1.95%
TF_2	*金秋九月, 丹桂飘香*。 [En el septiembre del otoño dorado, estamos rodeados por el osmanthus fragante.]	10	6	1	0	3	6	1.88%
TN_6	*在最后这不到一个月的时间里*, 中方将与参会各方保持密切沟通协调, *确保论坛顺利圆满举行, 推动论坛取得更多丰硕成果, 助力“一带一路”国际合作开辟新局面, 迈上新台阶*! [En menos de un mes, China mantendrá una estrecha comunicación y coordinación con todos los participantes para garantizar que el foro se celebre sin problemas y con éxitos, y promoverá que el foro logre resultados más fructíferos, contribuyendo a que la cooperación internacional de la Franja y la Ruta abra una nueva situación y suba a un nuevo escalón.]	79	51	3	0	51	51	1.72%
TE_4	*志合者, 不以山海为远*。 [Los que comparten el mismo ideal no se alejan debido a las distancias geográficas.]	11	10	1	0	10	10	1.70%
TP_5	双边贸易额*较建交之初增长了250多倍*, *达世界五分之一*。 [El volumen de comercio bilateral se ha incrementado en más de 250 veces en comparación con el inicio del establecimiento de relaciones diplomáticas, alcanzando una quinta parte del mundo.]	27	17	1	0	7	17	1.53%
TD_4	在今年的服贸会上, 覆盖服务贸易全部12大领域的展览, *展示了*中国服务贸易行业的繁荣全貌。 [En la Feria de Servicios de este año, las exhibiciones que cubren los 12 campos del comercio de servicios mostraron la prosperidad panorámica de la industria del comercio de servicios de China.]	43	26	0	2	9	26	1.37%
TL_9	近两年的*国际形势以及英国重新开放PSW签证, 学制短等优势*, *均导致*倾向英国留学的群体占比上升。 [Las ventajas del Reino Unido, como la situación internacional en los últimos dos años, la reapertura de visas PSW y el sistema escolar de corta duración, han llevado a un aumento en la proporción de grupos que tienden a estudiar en este país.]	46	28	1	1	17	28	1.30%
TF_4	在此我代表中央广播电视总台向远道而来的*各位嘉宾*表示诚挚的欢迎! [Aquí, en nombre del China Media Group, me gustaría expresar la bienvenida más cordial a todos los invitados que han venido desde lejos.]	31	18	2	0	19	18	1.21%
TP_3	建交40多年来, 经过双方几代人的共同努力, 中美关系成为世界上*相互交融最深, 合作领域最广, 共同利益最大*的双边关系之一。 [Desde el establecimiento de relaciones diplomáticas hace más de 40 años, gracias al esfuerzo conjunto de varias generaciones de ambas partes, las relaciones entre China y Estados Unidos se han convertido en una de las relaciones bilaterales con la integración mutua más profunda, los campos de cooperación más amplios y los intereses comunes más grandes del mundo.]	58	40	4	0	24	40	1.12%
TR_6	*今天的开幕式就到这里*。 [Ahora damos por concluida la ceremonia de apertura de hoy.]	11	7	0	0	4	7	1.07%
TQ_10	全球事务中许多其他领域*也是如此, 需要欧洲和中国携手合作*。 [Lo mismo ocurre en muchas otras áreas de los asuntos globales, que requieren la cooperación entre Europa y China.]	28	17	0	0	11	17	0.99%
TO_6	预计到2020年年底, *节能环保, 新能源装备, 新能源汽车等绿色低碳产业总产值将突破10万亿元人民币*。 [Se estima que para el final de 2020, el valor de producción total de la industria verde baja en carbono, como el ahorro energético y la protección medioambiental, las instalaciones de la nueva energía, los vehículos de nueva energía, etc., superará los 10 billones de yuanes.]	49	26	2	2	7	26	0.95%
TM_8	这种不断推动亚太开放, 合作, 共同发展的*动态进程*, *才是真正意义所*在。 [Este proceso dinámico de promover continuamente la apertura, la cooperación y el desarrollo común de Asia-Pacífico es donde reside el sentido real.]	33	21	0	0	12	21	0.93%

SYER, syntactic error rate.

Second, the complexity of sentence structures is another source of errors, due to factors including sentence length, numerous semantic roles, and dependency relationships, like in sentences TN_6, TP_5, TD_4, TL_9, TP_3 and TQ_6. In TN_6, the syntactic errors occur in the adverbial clause “在最后这不到一个月的时间里” [En este último tiempo de menos de un mes], for its high dependency relationship and the need to adjust word order due to differences between the two languages: “En este tiempo que menos de un mes” (在这不到一个月的时间里) (ST1467), “En los últimos tiempos, no alcanza un mes” (在最近不到一个月的时间里) (ST1472), “Durante los últimos tiempos que de menos de un mes” (在最近不到一个月的时间里) (ST1479), “En el/este último mes” (在过去这一个月里) (ST1476, 1,469, 1,473), “En la última etapa en que no llega un mes” (在不到一个月的最后阶段) (ST1477). Moreover, in TN_6, “确保论坛顺利圆满举行, 推动论坛取得更多丰硕成果, 助力“一带一路”国际合作开辟新局面, 迈上新台阶!” [Asegurar que el foro se lleve a cabo de manera fluida y exitosa, promover que el foro logre resultados más fructíferos y contribuir a abrir una nueva etapa y alcanzar un nuevo nivel en la cooperación internacional de la Franja y la Ruta.] is a serial verb construction in Chinese, which creates an additional challenge for the translator.

*En el último tiempo de menos de un mes, la parte china va a comunicar y coordinar estrechamente con todas partes del foro para que cumpla de manera viento en popa y impulsar el foro y gane más resultados y abrir una nueva etapa hacia una escala nueva.* (ST1470)

(*在不到一个月的最后时间里, 中方将与论坛各方密切沟通协调, 使论坛顺利举行, 推动论坛取得更多成果, 开启迈向新台阶的新阶段。*)

*En el último mes, China irá a mantener estrechos contactos con todos las partes que participan en la reunión y la cumbre, irá a impulsar el cumbre ganar más y impulsará la Franja y la Ruta.* (ST1469)

(*在过去一个月里, 中国将与参加会议和峰会的各方保持密切接触, 推动峰会取得更多成果, 并推进“一带一路”建设。*)

The syntactic complexity and unfamiliarity with Spanish grammar lead the ST1470 and the ST1469 to produce syntactic calques, failing to capture the syntactic logic and accurately reconstruct the sentence. Syntactic calques of the Chinese structure especially occur in sentences with relatively high dependency rate, or in run-on sentence. In TP_3, the interpretation “una de las relaciones bilaterles más que se ha armoniza profundamente en el ámbito de cooperación más grande y tiene el beneficio mutuo también más grande.” (其中一个最为和谐的双边关系, 在最广泛的合作领域中深入发展, 并具有最大的互利效益。) (ST1733) commits errors of awkward phrasing and incorrect word order.

In TP_5, errors are detected in the use of specific syntactic structures, particularly in comparative constructions:

*El comercio bilateral entre ambos países ha aumentado casi 250 veces al tiempo que recién establecido la relación diplomática, que ocupa casi un quinto parte de lo mundial.* (ST1756)

(*两国双边贸易额较建交之初增长了近250倍, 占全球贸易的近五分之一。*)

*El comercio bilateral múltiple 250 que el principio de la establecimiento de las relaciones que casi es uno por cinco en el mundo.* (ST1762)

(*双边贸易额是关系创建初期的250倍, 约占全球贸易的五分之一。*)

These errors clearly stem from using the comparative structure, especially in a sentence with a high dependency value (27), which reflects students’ inability to transfer complex Chinese sentences to a target language of the Spanish norms. This also supports the findings of several studies suggesting a greater cognitive load posed by sentences that need to be syntactically restructured in the target language (Gernsbacher and Shlesinger, 1997; Ma et al., 2021; Viezzi, 1989). Due to the differences in word order between Spanish and Chinese, the interpreters must rearrange the sentence elements to conform to the Spanish syntactic norms while preserving the original meaning, thus increasing the coordination effort (Chernovaty et al., 2023; Lee, 2012).

Apart from long and complex sentences, problems also occur in the distortion of syntactic structures. In the corpus, “在中国抗击疫情最困难的时刻” [en el momento más difícil de la lucha de China contra la pandemia] (TQ_9, SenT. Len 59, Dep. Rel 37, SYER 0.47%) is interpreted as “Cuando en el momento más difícil en China” (在中国最困难的时刻里) (ST1733) with the redundancy of “cuando” and “en.”

Regarding the sublevel of syntactic errors, apart from the already discussed substitution, omission is another serious syntactic error manifestation, that may occur when students encounter time constraints, complex sentence structures, or a substantial volume of information. Sentences TF_4, TR_6 in Table 7 have a high ER primarily due to partial or complete omissions. Their positions within the speech texts led to inappropriate attention allocation by the students. Specifically, TF_4 follows a lengthy sentence, while TR_6 is situated in the concluding section of the speech. The cognitive pressure experienced by interpreters appears to have contributed to them overlooking these sentences.

In summary, the sentences that exhibit higher SYER include poems, ancient saying, and sentences of long and complex structures. The predominant errors observed were substitutions related to sentence structure and omissions of syntactic elements. Additionally, lower proficiency in mastering various sentence structures and the usage of connecting words in Spanish hindered the translators’ ability to adjust sentence structures effectively. The causes of syntactic errors in interpretation are mainly the cognitive load caused by complicated and lengthy sentences, the differences in syntactic structure between Spanish and Chinese and the difficulty of adjusting the sentence structure, and the difficulty in using appropriate connectors between clauses. This result is also in accordance with previous studies that pointed out that difficult words and complex sentences cause increased cognitive effort (Shreve et al., 2010) and dysfluency (Shreve et al., 2011) in sight translation.

At the grammatical level, we detected the 10% sentences with the highest grammatical error rate (GER), as shown in Table 8. The results indicate that the sentences with the most grammatical errors are not necessarily those with relevant length or dependency relationship, but sentences that have different grammatical uses between Chinese and Spanish, like TC_3, TQ_2, TQ_1, TQ_7. Spanish verbal inflections, particularly in terms of tense and mood, represent grammatical features that are absent in Chinese, thus frequently leading to translation errors. For example, TC_3 has a high GER due to the verb tense. Students used the present tense for “强调” [enfatizar] (ST1761, 1758, 1755) and “应该” [deber] (ST1751, 1749, 1733, 1762, 1748, 1743, 1754), which is a common error of verb tense for Chinese students. In TQ_2, we detected errors of using the wrong tense when translating “期盼” [esperar], where the present perfect tense should have been used.

**Table 8:** The 10% of sentences with the highest grammatical error rates and their textual characteristics.

Sent ID	Source text sentence	Sent Len	Token	4CW	Trm	Sem. Rol	Dep. Rel	GER
TC_3	习近平*强调*, 科学技术*应该*造福全人类。 [Xi Jinping enfatizó que la ciencia y la tecnología deberían beneficiar a toda la humanidad.]	18	10	1	0	6	10	3.70%
TD_6	服贸会上的新技术, 新业态*吸引着*外媒的关注。 [Las nuevas tecnologías y nuevos formatos en la Feria de Servicios han atraído la atención de los medios extranjeros.]	21	14	1	1	5	14	3.64%
TQ_2	对于这场重要会晤, 各方*期盼已久*。 [Todas las partes han esperado por mucho tiempo con interés esta importante reunión.]	16	11	0	0	6	11	3.13%
TG_2	10月份, 生产*稳中有升*, 需求*企稳回暖*, 就业*继续改善*, *物价总体平稳*, *市场预期向好*, 国民经济运行*延续稳定恢复态势*。 [En octubre, la producción aumentó de manera constante, la demanda se estabilizó y repuntó, el empleo continuó mejorando, los precios en general se mantuvieron estables, las expectativas del mercado mejoraron y la economía nacional continuó recuperándose de manera estable.]	55	29	3	0	20	29	2.89%
TD_3	据统计, 本届服贸会吸引了包括*近200家世界500强企业*在内, 来自*148*个国家和地区的2.2万家企业和机构线上线下参展参会。 [Según las estadísticas, esta feria de servicios ha atraído a 22.000 empresas e instituciones de 148 países y regiones, incluidas casi 200 empresas de Fortune 500, para participar en línea y fuera de línea a esta exposición.]	61	35	0	2	11	35	2.60%
TQ_1	*中国, 德国, 欧盟领导人*将于*9月14日*通过视频方式*举行会晤*。 [Los líderes de China, Alemania y la Unión Europea se reunirán por video el 14 de septiembre.]	29	17	0	1	12	17	2.30%
TQ_7	面对巨大困难, *中国与欧盟密切合作*, *互帮互助*。 [Ante enormes dificultades, China y la Unión Europea han trabajado estrechamente para ayudarse mutuamente.]	22	13	0	2	12	13	2.27%
TP_5	双边贸易额较*建交*之初增长了*250多倍*, *达世界五分之一*。 [El volumen de comercio bilateral se ha incrementado en más de 250 veces en comparación con el inicio del establecimiento de relaciones diplomáticas, alcanzando una quinta parte del mundo,]	27	17	1	0	7	17	2.18%
TR_2	近年来, 在双方的*共同努力*下, 高层互访频繁, *人员往来*密切, 在经贸, 文化, 旅游, 教育等*各领域合作*不断深化, 友城关系呈现出蓬勃发展的良好态势。 [En los últimos años, gracias a los esfuerzos conjuntos de ambas partes, las visitas de alto nivel han sido frecuentes, los intercambios de personal han sido estrechos, se ha profundizado la cooperación en diversos campos como la economía y el comercio, la cultura, el turismo y la educación, y las relaciones de amistad-ciudad han mostrado un vigoroso desarrollo.]	68	42	2	0	22	42	2.16%
TR_3	为进一步夯实友好关系, 落实*双方*签订的年度合作协议, 促进双方政府和企业间的务实合作和*友好交流*, 今天, *甘肃省政府*和*纳瓦拉自治区政府*在这里*共同举办*友好合作交流会。 [Para consolidar aún más las relaciones amistosas, implementar el acuerdo de cooperación anual firmado por las dos partes y promover la cooperación pragmática y los intercambios amistosos entre los gobiernos y empresas, hoy, el gobierno de la provincia de Gansu y el Gobierno de la Comunidad Autónoma de Navarra están aquí para organizar conjuntamente un el foro de cooperación amistosa.]	79	43	4	2	25	43	2.08%
TF_6	共建“一带一路”, *秉持丝路精神*, 就是要倡导*开放包容, 互学互鉴*。 [Construir juntos la “Franja y la Ruta,” defender el espíritu de la Ruta de la Seda, es promover la apertura, la inclusión y el aprendizaje mutuo.]	31	20	3	0	9	20	2.02%
TQ_4	当前, 经济全球化遭遇困难, *单边主义, 保护主义抬头*, *世界面临的不稳定性, 不确定性*明显增多。 [El unilateralismo y el proteccionismo van en aumento, y el mundo se enfrenta a un marcado aumento de inestabilidad e incertidumbre.]	31	14	4	2	9	14	1.97%
TI_7	央行在专栏“发达经济体货币政策调整及应对”中*指出*, 近期主要发达经济体*货币政策*开始调整, 总的方向是边际收紧。 [El banco central señaló en la columna “Ajustes y respuestas de la política monetaria de las economías avanzadas” que las políticas monetarias de las principales economías avanzadas han comenzado a ajustarse recientemente y que la dirección general es un endurecimiento marginal.]	53	31	2	3	16	31	1.89%
TC_7	*希望*大会*围绕*“创新赋能可持续发展”这一主题, 集思广益, *增进*共识, *促进*合作, 使科技创新更好造福各国人民。 [Se espera que la conferencia se centre en el tema “La innovación potencia el desarrollo sostenible,” haciendo una lluvia de ideas, mejorando el consenso y promoviendo la cooperación, de modo que la innovación tecnológica pueda beneficiar mejor a las personas de todos los países.]	51	33	3	0	29	33	1.85%

GER, grammatical error rate.

*Es una conferencia muy importante que todas las partes la esperan por mucho tiempo.* (ST1751)

(*这是一个非常重要的会议, 各方期待很长时间。*)

*Todas las partes esperaban mucho esta importante reunión.* (ST1756)

(所有各方都非常期待这次重要会议。)

Moreover, for TQ_2, errors in subject-verb agreement were also detected.

Todas (*las) partes *ha esperado mucho.* (ST1745)

(所有各方已经期待了很长时间。)

*Y sobre esta importante conferencia, las partes ya he esperado por mucho tiempo.* (ST1743)

(关于这次重要会议, 各方已经期待了很长时间。)

TQ_1 includes errors regarding expressing dates, like “en (*el) 14 de septiembre” (9月14日) (ST1758), as well as subject-verb agreement errors, as in “Los dirigentes de China, Alemania y la Unión Europea se entrevistará” (中国, 德国和欧盟的领导人将进行会晤。) (ST1761), “Los líderes de China, Alemania y la Unión Europea se está reuniendo” (中国, 德国和欧盟的领导人正在举行会晤。) (ST1748), “los líderes de China, Alemania y la Unión Europea va a organizar una conferencia” (中国, 德国和欧盟的领导人将组织一次会议。) (ST1743), and using the simple past tense “se reunieron” (会晤) (ST1746) in the place where the future tense should have been used. TQ_7 shows the errors in determining the right verb tense and translating the predicate “密切合作, 互帮互助” [han trabajado estrechamente para ayudarse mutuamente] resulting in errors, such as:

*China y la Unión Europea van a cooperar mutuamente para ayudarse.* (ST1756)

(中国和欧盟将相互合作以互相帮助。)

*China y la Unión Europea ha hecho una cooperación estrecha y se han ayudado entre sí.* (ST1761)

(中国和欧盟已经进行了密切合作并相互帮助。)

*China y la UE ha trabajaban estrechamente para ayudarnos mutuamente.* (ST1750)

(中国和欧盟已经密切合作以相互帮助。)

TD_6 is an example of errors from using verb tense “estarán atrayendo a la atención” (将吸引关注) (ST1754) or subject-verb agreement in interpreting “吸引着外媒的关注” [han atraído la atención de los medios extranjeros], like “ha atraído la atención” (吸引着外媒的关注) (ST1756, 1745, 1747). Although the structure of the Chinese sentences is not inherently complex, the fundamental differences between Chinese and Spanish grammar are the main cause of these errors. Moreover, insufficiency in linguistic and formal schemata of the target language also contribute to such errors.

Furthermore, the sentences with significant length and dependency relationship have a tendency of exhibiting more errors, like TG_2, TR_2, TR_3, TI_7, TC_7, due to the bigger memory and coordination pressure. Take the sentence TG_2 for example:

*Ha demostrado que, en el mes pasado, las producciones interiores ha registrado muchos aumentos cuando mantenía su estado estable y así como las demandas con uno repuntes importantes y se sigue mejorándose las condiciones del empleo y los precios sigue con su nivel generalmente estable. Con todo eso se prevé una perspectiva muy buena y positiva del mercado y el funcionamiento de la economía nacional ha persistido hacia su rumbo de restauración y de manera muy estable.* (ST1751)

(已证明, 在过去一个月中, 国内生产稳中有升, 需求也出现了重要回升, 就业条件持续改善, 价格总体保持稳定水平。综上所述, 市场前景非常积极乐观, 国民经济运行延续稳定恢复态势。)

ST1751 makes errors in subject-predicate agreement due to the length of the sentence, therefore increasing the coordination pressure. In the TD_3 of ST1743, we detected the errors of using the past participle form “incluido” (包括) of the verb “incluir” (包括) instead of using its gerund form “incluyendo” (包括). There are also errors of agreement in number and gender in the number “doscientos” (二百), and in the verb tense of “participar” (参加).

*Según el diálogo y según los datos, esta feria ha atraído, incluido casi doscientos de las empresas Fortune Global 500, mientras incluido 148 países y regiones de casi 22,000 empresas y institutos que participan a la feria de manera virtual o fiscal.* (ST1743)

(根据对话和数据, 这次交易会吸引了包括近200家全球500强企业, 以及148个国家和地区的近22,000家企业和研究所线上线下参与。)

In TI_7 of ST1683, we detected errors of the verb tense of “señala” (指出), where “señalado” (指出的) should be used, as well as erroneous use of “la política monetaria” (货币政策), where the plural form should be employed:

*El banco central señala en la columna “Ajustes y respuestas de la política monetaria de las economías avanzadas” que la política monetaria de las principales economías avanzadas han comenzado a ajustarse recientemente con la dirección general de estricción marginal.* (ST1683)

(央行在专栏“发达经济体货币政策调整及应对”中指出, 近期主要发达经济体的货币政策已开始调整, 总的方向是边际收紧。)

These errors are caused due to the complexity of tense conjugation, the voice and mood of Spanish verbs, and the difficulty of identify the subject in a long sentence. The insufficiency of grasping the grammatical rules of Spanish is also a cause of errors. Chinese lacks grammatical gender and a robust system of noun-adjective agreement based on number. This fundamental difference in language structure makes it challenging for Chinese learners to internalize the Spanish system of grammatical gender and number agreement. Another possible error cause is that the students prioritize conveying meaning, which, however, could deviate their attention from the grammar in long and complex sentences.

Regarding the manifestation of grammatical errors, as shown in Table 4, the most prominent sublevel of error is the agreement between gender and number, manifested in various forms. Moreover, this sublevel of errors is not concentrated on specific expressions or grammatical structures, but rather spread across a variety of linguistic contexts. For example, we detected the following errors in sentence TR_2:

*Como la provincia Gansu y la Autónoma Española Navarra se establecieron en 2005 relación de amistad, que en los últimos años, con el esfuerzos comunes de ambos partes, el intercambio de alto nivel está con más frecuencias, y como así de los intercambios personales, y las cooperaciones en todas las áreas como la comercial, cultural, del turismo y de educación serán más profunda.* (ST1756)

(由于2005年甘肃省与西班牙纳瓦拉自治区建立友好关系, 近年来通过双方的共同努力, 高级别交流更加频繁, 人员交流亦是如此, 商业, 文化, 旅游和教育等领域的合作将更加深入。)

*En los últimos años, gracias a los esfuerzos conjunto de ambas partes, las visita de alto nivel ha sido frecuentes …* (ST1754)

(*近年来, 由于双方的共同努力, 高级别访问频繁 …*)

From the errors, we can see that the forms vary from gender agreement of articles and adjectives “el esfuerzos comunes” (共同努力) and “las visita” (访问), to errors in number agreement within noun phrases, such as “ambos partes” (双方) and “los esfuerzos conjunto” (共同努力). Agreement errors may also be generated because of the source text influence and short-memory effort failure in long sentences.

Errors in verb tense and conjugation, inconsistency in verb tense, and in singular and plural verbs and in subject-predicate are also prevalent, as discussed in the previous examples. Prepositions and articles are two grammatical phenomenon that do not occupy a high percentage of errors, but worth noticing that within these two sublevels, the most frequent errors are substitution of preposition, and omission and addition of articles. The cause of the prepositional errors could be interference from the mother tongue, as Chinese and Spanish have very different prepositional systems. Spanish has a wide range of prepositions, each with specific uses and meanings. Some prepositions can be used interchangeably in certain contexts, whereas others have very distinct uses. This complexity can be challenging for Chinese interpreters to grasp fully. Some prepositional errors may arise from a lack of in-depth understanding of the Spanish language’s grammatical structures and idiomatic expressions.

Regarding the errors in articles, omission and addition were the main error manifestations. An obvious error is the omission of definite articles, such as “invitar a (*el) Sr. Zhang” (有请张先生) (TR_7, ST1754), “Los líderes de China, Alemania, (*la) Unión Europea” (中国, 德国, 欧盟的领导人) (TQ_1, ST1746) “reunirse en (*el) 14 de septiembre” (9月14日会晤) (TQ_1, ST1758), and “el intercambio de (*el) personal” (人员交流) (TP_6, ST1733). The addition occurs when using vocative case, such as “Estimados todos, los líderes, los invitados, los maestros y los alumnos” (各位领导, 各位来宾, 老师们, 同学们) (TB_1, ST1756); or adding articles before a proper noun, for example, “la Europa y China” (欧洲和中国) (TQ_10, ST1743).

We can see that grammatical errors made by students are predominantly concentrated in areas where Spanish grammar differs from or lacks direct equivalence to Chinese grammar, such as agreement in gender and number, verb tense and conjugation, prepositions, and articles. These errors are also typical difficulties for Chinese learners of Spanish, and they are highlighted in the Chinese–Spanish interpretations. This finding is in line with Agrifoglio (2004), who found that interpreters often lost the referent and forgot the gender, number, and person. This problem may have been exacerbated by the fact that inflection is more common in Spanish and that adjectives, pronouns, and verbs need to be put into proper agreement.

### 5.2 Error manifestation

The error distribution and frequency analysis show that students have major problems correctly using Spanish expressions, whereas errors of redundancy and omissions are not as serious (see Table 4). This result is in accordance with the discovery by Agrifoglio (2004), who argues that sight translation has an advantage over simultaneous interpretation and consecutive interpretation in the content, thus committing fewer errors related to the content, such as omissions, changes in meaning, or incomplete sentences, but more errors in expression, such as lexical, syntactic, or grammatical problems, calques, and language style, mostly caused by short-term memory failures of retrieving information from the beginning of sentences, or the formulation the interpreter has already embarked on, especially where grammatical structures differ markedly between the two languages.

Table 9 shows that sentences that frequently contain substitution errors also appear to have higher error rate at the lexical, syntactic or grammatical levels (refer to Tables 6–8). These sentences have complex structures or high dependency relationships (see Table 10, TP_5, TD_3, TC_7, TL_9), include terminology or neologisms that students have not yet mastered (TD_6, TG_3, TD_5, TD_4), use four-character or abstract words with difficult-to-find Spanish equivalents, or exhibit more than one of these characteristics (TL_9). This phenomenon is also explained by the high percentage of substitution at each of the three error levels and in the total count of the corpus (Table 4). Therefore, the high percentage of substitution errors at all levels suggests a possible correlation between substitution error rate (Sub. ER) and error concentration in sentences (Table 9), though further investigation may be needed to confirm this relationship.

**Table 9:** Error rates of the 10% of sentences with the highest Sub. ER.

Sentence ID	LER	SYER	GER	Sub. ER	Add. ER	Omi. ER	Sent. ER
TD_6	3.92%	0.28%	3.64%	**6.72%**	0.00%	1.12%	7.56%
TP_5	2.61%	1.53%	2.18%	**6.32%**	0.00%	0.00%	6.32%
TD_3	4.15%	0.87%	2.60%	**6.27%**	0.10%	1.25%	7.62%
TG_3	4.73%	0.00%	1.15%	**5.74%**	0.00%	0.14%	5.88%
TD_5	5.50%	0.19%	1.33%	**5.69%**	0.00%	1.33%	6.45%
TQ_2	2.43%	0.35%	3.13%	**5.21%**	0.35%	0.35%	5.90%
TR_5	5.88%	0.39%	1.18%	**5.10%**	0.39%	1.96%	7.45%
TD_4	4.79%	1.37%	1.23%	**5.06%**	0.14%	2.19%	7.39%
TC_7	3.27%	0.76%	1.85%	**5.01%**	0.00%	0.87%	5.88%
TN_1	4.15%	0.83%	0.50%	**4.98%**	0.00%	0.50%	5.48%
TF_6	2.62%	0.60%	2.02%	**4.84%**	0.20%	0.20%	5.24%
TN_2	4.17%	0.00%	0.89%	**4.76%**	0.00%	0.30%	5.06%
TC_3	0.93%	0.31%	3.70%	**4.63%**	0.00%	0.31%	4.94%
TL_9	2.75%	1.30%	0.87%	**4.49%**	0.00%	0.43%	4.93%

Sub. ER, substitution error rate; Add. ER, addition error rate; Omi. ER, omission error rate; Sent. ER, sentence error rate.

**Table 10:** The 10% of sentences with the highest substitution error rates.

Sent ID	Source text sentence	Sent Len	Token	4CW	Trm	Sem. Rol	Dep. Rel	Sub. ER
TD_6	*服贸会*上的*新技术, 新业态*吸引着外媒的关注。 [Las nuevas tecnologías y nuevos formatos en la Feria de Servicios han atraído la atención de los medios extranjeros.]	21	14	1	1	5	14	6.72%
TP_5	双边贸易额较建交之初*增长了250多倍*, 达世界*五分之一*。 [El volumen de comercio bilateral se ha incrementado en más de 250 veces en comparación con el inicio del establecimiento de relaciones diplomáticas, alcanzando una quinta parte del mundo.]	27	17	1	0	7	*17*	6.32%
TD_3	据统计, 本届服贸会吸引了包括近*200家世界500强企业*在内, 来自*148个国家和地区的2.2万家企业和机构*线上线下参展参会。 [Según las estadísticas, esta feria de servicios ha atraído a 22.000 empresas e instituciones de 148 países y regiones, incluidas casi 200 empresas de Fortune 500, para participar en línea y fuera de línea a esta exposición.]	61	35	0	2	11	*35*	6.27%
TG_3	“总的来看, 10月份*国民经济*持续*恢复*增长, *‘六稳”六保’*任务落实成效进一步显现。” [“En general, la economía nacional continuó reanudando su crecimiento en octubre, y la implementación de las tareas de ‘seis estabilidad’ y ‘seis garantías’ ha demostrado aún más su efectividad”.]	41	22	2	1	10	22	5.74%
TD_5	*数字贸易, 5G通讯, 工业互联网, 智慧办剬, 区块链创新, 跨境电商*…… [Comercio digital, comunicaciones 5G, Internet industrial, oficina inteligente, innovación blockchain, comercio electrónico transfronterizo …]	31	21	0	*6*	0	21	5.69%
TQ_2	*对于*这场重要*会晤*, 各方*期盼已久*。 [Todas las partes han esperado por mucho tiempo con interés esta importante reunión.]	16	11	0	0	6	11	5.21%
TR_5	向对本次活动*给予*大力支持的*相关单位*和参会企业表示*衷心的感谢*! [También me gustaría agradecer a las instituciones concernientes y las empresas participantes que han brindado un fuerte apoyo a este evento.]	30	19	0	0	18	19	5.10%
TD_4	在今年的服贸会上, 覆盖服务贸易全部12大*领域*的展览, 展示了中国服务贸易行业的繁荣*全貌*。 [En la Feria de Servicios de este año, las exhibiciones que cubren los 12 campos del comercio de servicios mostraron la prosperidad panorámica de la industria del comercio de servicios de China.]	43	26	0	2	9	26	5.06%
TC_7	*希望*大会*围绕*“*创新赋能可持续发展*”这一主题, *集思广益*, 增进共识, 促进合作, 使科技创新更好*造福*各国人民。 [Se espera que la conferencia se centre en el tema “La innovación potencia el desarrollo sostenible,” haciendo una lluvia de ideas, mejorando el consenso y promoviendo la cooperación, de modo que la innovación tecnológica pueda beneficiar mejor a las personas de todos los países.]	51	33	3	0	29	*33*	5.01%
TN_1	我们*期待*, 以*开放包容*, 合作共赢理念为引领, 推动构建*更加剬正, 合理和均衡的全球治理体系*。 [Esperamos construir un sistema de gobernanza global más justo, razonable y equilibrado guiado por el concepto de apertura, inclusión y cooperación de beneficio mutuo.]	43	26	2	1	19	26	4.98%
TF_6	共建“一带一路”, *秉持*丝路精神, 就是要*倡导开放包容, 互学互鉴*。 [Construir juntos la Franja y la Ruta, defender el espíritu de la Ruta de la Seda, es promover la apertura, la inclusión y el aprendizaje mutuo.]	31	20	3	0	9	20	4.84%
TN_2	*开放包容, 合作共赢*是“*一带一路*”倡议的*核心理念*。 [La apertura, la inclusión y la cooperación de beneficio mutuo son los conceptos nucleares de la iniciativa la Franja y la Ruta.]	24	16	3	0	3	16	4.76%
TC_3	习近平*强调*, 科学技术*应该*造福全人类。 [Xi Jinping enfatizó que la ciencia y la tecnología deberían beneficiar a toda la humanidad.]	18	10	1	0	6	10	4.63%
TL_9	近两年的国际形势以及*英国*重新开放*PSW签证*, *学制*短等优势, *均导致*倾向英国*留学的群体*占比上升。 [Las ventajas del Reino Unido, como la situación internacional en los últimos dos años, la reapertura de visas PSW y el sistema escolar de corta duración, han llevado a un aumento en la proporción de grupos que tienden a estudiar en este país.]	46	28	1	1	17	*28*	4.49%

The second common form of error is omission (Table 11), as students tend to omit words in certain professional areas, terminology, poetic and expressions (TF_2, TE_4, TB_4), among others, which they do not know how to express in Spanish. For example, in TO_5, professional terms, like “增加值” [valor añadido] (ST1751, 1746), are omitted, as well as numerical expressions “15%左右” [cerca de 15%] (ST1751, 1758), and the adverbial clause “近年来” [en los últimos años] (ST1756, 1746). In TD_4, “全貌” [el panorama] is omitted because the students do not know how to express it in Spanish (ST1756, 1749, 1733, 1770, 1748, 1747, 1743, 1754). “相关单位” [entidades concernientes] in TR_5 (ST1756, 1733, 1760, 1748) and the neologism of “新业态” [el nuevo formato] in TM_4 (ST1733, 1762, 1760, 1748, 1757, 1746, 1753) are omitted for the same reason.

**Table 11:** The 10% of sentences with the highest omission error rates and their textual characteristics.

Sent ID	Source text sentence	Sen Len	Token	4CW	Trm	Sem. Rol	Dep. Rel	Omi. ER
TD_2	本届服贸会, 是*新冠肺炎疫情发生以来*, 中国在*线下*举办的第一场, 也是全球第一场*重大*国际*经贸*活动。 [Esta feria de servicios es el primer evento fuera de línea que se realiza en China desde el estallido de la nueva epidemia de neumonía de la corona virus, y también es el primer evento económico y comercial internacional importante del mundo.]	46	30	0	1	17	30	2.43%
TO_5	国家节能中心副主任杨博表示, *近年来*中国节能环保产业*增加值*以年均*15%左右*的速度增长。 [Yang Bo, subdirector del Centro Nacional de Ahorro Energético, dijo que en los últimos años, el valor agregado de la industria de ahorro energético y protección ambiental de China ha aumentado a una tasa anual promedio de alrededor de 15%.]	42	22	1	1	7	22	2.22%
TL_2	又有哪些新的变化? [¿Cuáles son los nuevos cambios?]	9	7	0	0	7	7	2.22%
TD_4	在今年的服贸会上, *覆盖服务贸易*全部12大领域的展览, 展示了中国服务贸易行业的繁荣*全貌*。 [En la Feria de Servicios de este año, las exhibiciones que cubren los 12 campos del comercio de servicios mostraron la prosperidad panorámica de la industria del comercio de servicios de China.]	43	26	0	2	9	26	2.19%
TR_5	向对本次活动给予大力支持的*相关单位*和参会企业表示*衷心的感谢*! [También me gustaría agradecer a las instituciones concernientes y las empresas participantes que han brindado un fuerte apoyo a este evento.]	30	19	0	0	18	*19*	1.96%
TF_2	*金秋*九月, *丹桂*飘香。 [En el septiembre del otoño dorado, estamos rodeados por el osmanthus fragante.]	10	6	1	0	3	6	1.88%
TM_4	比如, 根据新愿景, 未来APEC将推动数字经济*新业态*新模式发展。 [Por ejemplo, de acuerdo con la nueva visión, APEC promoverá el desarrollo de nuevos formatos y modelos de la economía digital en el futuro.]	31	18	0	2	7	18	1.74%
TE_4	*志合者, 不以山海为远*。 [Los que comparten el mismo ideal no se alejan debido a las distancias geográficas.]	11	10	1	0	10	10	1.70%
TP_6	双向投资从*几乎为零*攀升到近2400亿*美元*, *每年*人员往来达500万*人次*。 [La inversión bidireccional ha aumentado de casi cero a casi 240 mil millones de dólares estadounidenses, y los intercambios anuales de personal alcanzan los 5 millones de personas veces.]	35	20	1	0	16	20	1.68%
TO_6	*预计到2020年年底*, 节能环保, 新能源装备, 新能源汽车等绿色低碳产业*总产值*将突破*10万亿元*人民币。 [Se estima que para el final de 2020, el valor de producción total de la industria verde baja en carbono, como el ahorro energético y la protección medioambiental, las instalaciones de la nueva energía, los vehículos de nueva energía, etc., superará los 10 billones de yuanes.]	49	26	2	2	7	26	1.63%
TB_4	五十年*栉风沐雨*不寻常, 五十年*辛勤耕耘*谱华章。 [Cincuenta años de arduo trabajo son inusuales, y componen un capítulo brillante.]	22	13	2	0	11	13	1.62%
TR_7	*下面进入交流会第二阶段, 有请省政府外事办主任张先生主持。 谢谢大家!* [Ahora entramos en la segunda etapa del foro, que será moderado por el Sr. Zhang, director de la Oficina de Relaciones Exteriores del Gobierno Provincial. ¡Gracias a todos!]	33	18	0	1	8	18	1.60%
TC_5	中国愿同各国一道, 加强科技创新与合作, 促进更加开放*包容*, *互惠共享*的国际*科技创新交流*, 为推动全球经济*复苏, 保障*人民身体健康作出贡献。 [China está dispuesta a trabajar con otros países para fortalecer la innovación y la cooperación tecnológica, promover intercambios internacionales de innovación tecnológica más abiertos, inclusivos y mutuamente beneficiosos, y contribuir a promover la recuperación económica mundial y garantizar la salud del pueblo.]	65	39	2	0	39	39	1.45%
TD_5	数字贸易, 5G通讯, 工业互联网, 智慧办剬, 区块链*创新*, *跨境电商*…… [Comercio digital, comunicaciones 5G, Internet industrial, oficina inteligente, innovación blockchain, comercio electrónico transfronterizo …]	31	21	0	6	0	21	1.33%

Moreover, students also omitted words in complex structure sentences, long sentences, or high dependency relationship, due to linguistic differences and memory and coordination pressure. For example, in TR_5, students (ST1750, 1751) omitted the object “感谢” [agradecimientos] probably due to the long adverbial clause and modifiers. In TD_3, which is another long sentence, students omitted “线上线下” [en línea y de forma presencial] (ST1756, 1733, 1748, 1746) or “参展参会” [participar en la exposición] (ST1756, 1746) because of the sentence length and the unfamiliarity with the equivalent Spanish expressions. There are also cases of omission of whole phrases. We detected that ST1754 and ST1746 omitted parts of the sentence TO_6 for evasion due to their lack of ability to translating the words and sentence structure.

In TR_7, a high omission error rate (Om. ER) is also detected, although it’s a sentence with simple structure and words. Two cases of omission of whole sentence are detected (ST1750, 1746), as well as one case of omission of the second part of the sentence “有请省政府外事办主任张先生主持。谢谢大家!” [Invitamos al señor Zhang, director de la Oficina de Asuntos Exteriores del Gobierno Provincial, a presidir. ¡Gracias a todos!] (ST1748). This phenomenon is unusual in the corpus, so we assume that the omission is caused by the concentration of attention in the core information of the text and the ignorance of the last sentence. Other omission errors of this sentence are related to the terminology, like interpreting “省政府” [la Oficina de Asuntos Exteriores del Gobierno Provincial] as “Oficina de los Asuntos Exteriores” (ST1762) or omitting “张先生” [el señor Zhang]. We assume that this omission is partly due to unfamiliarity of expression of Chinese institutions in Spanish, but the main cause of error should be the pressure interpreter feels and the attention allocation issue.

In conclusion, the textual characteristics of sentences that concentrate errors of omission can be terminological or poetic expressions, or other words that are difficult to translate, leading to students omitting information for evasion. Omission can also be caused by cognitive load or attention allocation issue.

Within addition, which is the third type of error manifestation, we firstly examined grammatical additions. In TB_1 and TR_7 (Table 12), students used articles in unnecessary positions, concretely speaking, before vocatives. The following are errors detected in TB_1:

**Table 12:** The 10% of sentences with the highest addition error rates and their textual characteristics.

Sent ID	Source text sentence	Sen Len	Token	4CW	Trm	Sem. Rol	Dep. Rel	Add. ER
TB_1	尊敬的*各位领导, 各位来宾, 老师们, 同学们*:大家上午好! [Estimados líderes, invitados, profesores y estudiantes: ¡Buenos días a todos!]	27	18	0	0	3	18	0.79%
TR_7	下面进入交流会第二阶段, 有请省政府外事办主任*张先生*主持。谢谢大家! [Ahora entramos en la segunda etapa del foro, que será moderado por el Sr. Zhang, director de la Oficina de Relaciones Exteriores del Gobierno Provincial.¡Gracias a todos!]	33	18	0	1	8	18	0.53%
TC_1	国家主席习近平10日向博鳌亚洲论坛国际科技与创新论坛*首届大会*开幕致贺信。 [El 10 de noviembre, el presidente Xi Jinping envió una carta de felicitación a la inauguración del primer Foro Internacional de Tecnología e Innovación de Boao de Asia.]	50	25	0	2	6	25	0.44%
TG_1	11月16日, 国家统计局发布了10月份*国民经济运行情况*。 [El 16 de noviembre, la Oficina Nacional de Estadísticas publicó la situación de operación económica nacional de octubre.]	28	15	1	1	6	15	0.42%
TQ_10	全球事务中许多*其他*领域也是如此, 需要*欧洲*和中国携手合作。 [Lo mismo ocurre en muchas otras áreas de los asuntos globales, que requieren la cooperación entre Europa y China.]	28	17	0	0	11	17	0.40%
TR_5	向对本次活动*给予大力支持*的相关单位和参会企业表示*衷心的感谢*! [También me gustaría agradecer a las instituciones concernientes y las empresas participantes que han brindado un fuerte apoyo a este evento.]	30	19	0	0	18	19	0.39%
TQ_1	中国, 德国, 欧盟领导人将于*9月14日*通过*视频方式*举行会晤。 [Los líderes de China, Alemania y la Unión Europea se reunirán por video el 14 de septiembre.]	29	17	0	1	12	17	0.38%
TQ_8	在中国抗击疫情最困难的时刻, 欧洲多国向中国*捐赠抗疫物资*, 欧洲民间也通过*音乐会, 义卖募捐*等多种形式为中国提供了宝贵支持。 [En el momento más difícil para China de combatir la epidemia, muchos países europeos donaron suministros anti-epidemia a China. Además, los pueblos europeos también brindaron un valioso apoyo a China a través de conciertos, ventas benéficas y otras formas.]	59	37	1	0	17	37	0.38%
TQ_2	对于这场重要会晤, *各方*期盼已久。 [Todas las partes han esperado por mucho tiempo con interés esta importante reunión.]	16	11	0	0	6	11	0.35%
TG_2	10月份, 生产稳中有升, 需求企稳回暖, 就业继续改善, 物价总体平稳, 市场预期*向好*, *国民经济运行延续稳定恢复态势*。 [En octubre, la producción aumentó de manera constante, la demanda se estabilizó y repuntó, el empleo continuó mejorando, los precios en general se mantuvieron estables, las expectativas del mercado mejoraron y la economía nacional continuó recuperándose de manera estable.]	55	29	3	0	20	29	0.32%
TQ_6	随着*新冠疫情*不断蔓延, 全球经济社会运转遭到严重冲击, 引发复杂, *动荡的*局面。 [A medida que la nueva epidemia de la corona continúa ampliándose, las operaciones económicas y las sociedades mundiales se han visto gravemente afectadas, provocando una situación compleja y turbulenta.]	37	21	2	0	13	21	0.30%
TR_3	为进一步*夯实友好关系*, 落实双方签订的年度合作协议, 促进双方政府和企业间的务实合作和友好交流. 今天, *甘肃省政府和纳瓦拉自治区政府*在这里共同举办友好合作交流会。 [Para consolidar aún más las relaciones amistosas, implementar el acuerdo de cooperación anual firmado por las dos partes y promover la cooperación pragmática y los intercambios amistosos entre los gobiernos y empresas, hoy, el gobierno de la provincia de Gansu y el Gobierno de la Comunidad Autónoma de Navarra están aquí para organizar conjuntamente un el foro de cooperación amistosa.]	*79*	43	4	2	25	*43*	0.30%
TO_6	预计到2020年年底, *节能环保, 新能源装备, 新能源汽车等*绿色低碳产业总产值将突破*10万亿元*人民币。 [Se estima que para el final de 2020, el valor de producción total de la industria verde baja en carbono, como el ahorro energético y la protección medioambiental, las instalaciones de la nueva energía, los vehículos de nueva energía, etc., superará los 10 billones de yuanes.]	49	26	2	2	7	26	0.27%
TF_7	各位嘉宾, 各位朋友!只要我们共同携手, 齐心协力, 就一定能*谱写出合作共赢道路上更加璀璨的华章*! [¡Distinguidos invitados y amigos! ¡Mientras trabajemos juntos y trabajemos juntos, seremos capaces de escribir un capítulo más brillante sobre el camino hacia la cooperación de beneficio mutuo!]	46	27	1	0	7	27	0.27%

*Estimados dirigentes, los invitados, profesores y estudiantes: ¡Buenos días!* (ST1756)

(尊敬的领导, 来宾, 老师和学生们: 早上好!)

*Distinguidos dirigentes, los invitados, los maestros y los estudiantes. Buenos días.* (ST1761)

(尊敬的领导, 来宾, 老师和学生们。早上好。)

*Estimados todos, los líderes, los invitados, los maestros y los alumnos.* (ST1747)

(尊敬的领导, 来宾, 老师和学生们。)

In TR_7, when interpreting “省政府外事办主任张先生” [el señor Zhang, director de la Oficina de Asuntos Exteriores del Gobierno Provincial], students add articles like “el director de la Oficina de Relaciones Exteriores del Gobierno Provincial” (省政府外事办主任) (ST1745), “el jefe de los Asuntos Extranjeros del Gobierno” (政府外事办主任) (ST1733) or “el director de los Asuntos Exteriores del Gobierno” (政府外事办主任) (ST1747). Addition of articles also appeared when listing, as in TQ_8:

*El pueblo europeo también ayuda mucho por diferentes maneras. Por ejemplo, los conciertos, las recaudaciones, etcétera.* (ST1753)

(欧洲人民也以不同方式提供了很多帮助。例如, 音乐会, 筹款等等。)

*Y en el momento más difícil en contra a la pandemia en China, muchos países de Europa donaron los suministros anti-epidemia a China.* (ST1747)

(在中国对抗疫情最困难的时刻, 许多欧洲国家向中国捐赠了抗疫物资。)

*Y en los momentos más difíciles de la guerra contra COVID-19 en China, muchos países europeos residieron a China suministros anti-epidemia. Y el pueblo europeo también apoyaron a China en muchas medidas, por ejemplo, los conciertos o los denoción.* (ST1743)

(在中国抗击COVID-19疫情最困难的时刻, 许多欧洲国家居住到中国了抗疫物资。欧洲人民也通过多种方法支持中国, 例如, 音乐会或捐款。)

In TF_7, we detected two cases of additional article use in four-character words, like “una historia espléndida de la cooperación y las ganancias compartidas” (合作与共赢的辉煌历史) (ST1762), and “capítulos más espléndidos en el camino de la cooperación y la ganancia mutua” (合作与共赢道路上更辉煌的篇章) (1761). Additions are also detected in expressions of dates, such as “en 14 de septiembre” (9月14日) (TQ_1, ST1733), and regarding countries, like “la Europa” (欧洲) (TQ_10, ST1743). Moreover, the addition of words occurred when the students failed to grasp the meaning or the correct use of certain words, adding unnecessary expressions. For example, when interpreting “大会” [congreso] in TC_1, students committed errors, like “la primera conferencia del foro” (论坛第一次会议) (ST1749), “la primera asamblea del foro” (论坛第一次大会) (ST1751), and “el primer congreso de foro” (论坛第一次代表大会) (ST1762). In TR_3, ST1762 added the information “los líderes” (领导), which is not mentioned in the source text:

*Para profundizar esta cooperación, y para aplicar los acuerdos suscritos anuales de ambas partes, y también para promover las cooperaciones prácticas y intercambio amistoso entre ambos gobiernos empresarias, hoy en día, los líderes de gobierno de la provincia de Gansu y la Comunidad Autónoma de Navarra va a organizar una conferencia de la cooperación amistosa aquí.* (ST1762)

(为了深化这一合作, 落实双方签署的年度协议, 并促进双方政府企业之间的务实合作和友好交流, 今天, 甘肃省政府和纳瓦拉自治区的政府领导人将在这里组织一场友好合作会议。)

The sentence length of TR_3 probably increases the cognitive load, as well as memory and coordination effort of the interpreter. Student may therefore add words without being able to memorize all the information.

To sum up, the addition occurs mainly at the grammatical and lexical levels. The diverse textual features of sentences with high addition error rate (Add. ER) indicate that the students are prone to making such errors across various text properties. Some sentences employ a more formal and elegant language style, for which the students often lack the sufficient Spanish expressions repertoire and therefore fail to express correctly. Addition of articles in Spanish is frequent, discussed in depth in the error level section, due to the grammatical differences between Chinese and Spanish. Word additions, on the other hand, stem from insufficient Spanish linguistic schemata at both the lexical and syntactic levels, resulting in heightened cognitive load and psychological pressure. We can see that the addition is committed mainly for lack of linguistic schemata. The addition of information is rare in the corpus, probably due to, to a large extent, the form of sight translation, which includes the continuous presence of the source-language text and easier monitoring of the output.

## 6 Conclusion

This study aimed to analyze the error distribution pattern in a Chinese–Spanish sight translation error corpus and, based on the error taxonomy that we formulated, and a combination of quantitative and qualitative analysis, determine interpretation problems of Chinese undergraduate student interpreters and find the error causes. According to the quantitative analysis of the textual characteristics of source texts, we found no significant correlation between the error levels or error manifestations across various articles. This suggests that translation themes and text genres are not decisive factors influencing error occurrence. Instead, the consistency of error types across different texts might imply that certain challenges are inherent to the translation process itself, irrespective of the content or genre. This finding also suggested the possibility of shared characteristics among translators, which could lead to similar error patterns regardless of the articles’ themes or styles.

In terms of error levels, we focused on the high ER sentences and prominent sublevel errors and their error causes. At the lexical level, sentences with abstract words, poetic expressions, terminologies or neologisms, and fixed collocations or four-character words, tend to have a higher LER. In error manifestation, substitution of word selection and terminology, and omission of words were most prominent. The error causes were semantic differences between Chinese and Spanish, and insufficient linguistic and cultural schemata can also lead to lexical errors. Problems manifested in terminologies highlight the importance of vocabulary and background information preparation prior to sight translation. At the syntactic level, sentences with poems and ancient saying, as well as those of complex sentence structure or specific Chinese structures, concentrated more errors. The most frequent error manifestations were substitution related to sentence structure and omission of syntactic elements. Prominent error causes at this level could include differences in syntactic structure between the source and target languages, leading to distortion of sentence structure and omission of syntactic elements. The sentence length, numerous dependency relationship and complex sentence structure could also increase the cognitive load, thus leading to more errors. At the grammatical level, errors were committed mainly in grammatical uses that differ markedly between Chinese and Spanish, like terms of agreement in gender and number, verb tense and conjugation, prepositions, and articles. Difficult words, complex sentence structures and sentence length can increase the cognitive load and coordination pressure and strengthen the negative transference, therefore hindering the interpreters’ performance.

Regarding error manifestation, substitution was the major error manifestation in the corpus, significantly surpassing addition and omission. This unbalanced error distribution can be attributed to the particularities of sight translation, in which the interpreters depend largely on the source text and commit less content-related errors and more errors in the expression. We believe that the abundant errors in expression are also caused by the specificity of the Chinese–Spanish language pair, which presents remarkable linguistic differences at all levels and cause negative mother language transference, therefore generating errors in the Spanish production. Moreover, the lack of domain knowledge and anxiety can also increase the cognitive load of the interpreters and cause more errors in the translation process. The low percentage of additions and omissions was partially due to the continuous presence of the source text. Furthermore, addition in translation refers to formal expansion rather than the incorporation of content not present in the source text, manifested mainly in addition of articles and words due to erroneous usage or understanding. Omissions are committed because of the students’ inability to interpret certain information and use of a strategy of evasion. In other cases, omissions appear due to cognitive load, psychological pressure and inappropriate attention allocation.

We believe that the present research contributes in several ways. By formulating an error taxonomy, the research provides a more systematic and structured theoretical framework for error identification and evaluation, interpreting quality control and interpreting studies. In this way, scholars can gain a deeper understanding of common error types and their causes in the interpreting process, and have the possibility of improving interpreting training in future work. A limitation of this study lies in the lack of participant-focused investigation due to time constraints. Future research could benefit from an in-depth participants examination through questionnaires or longitudinal studies exploring the relationships between their language proficiency, interpretation competence, psychological states, and interpreting quality.

## Data Availability

The raw data supporting the conclusions of this article will be made available by the authors without undue reservation.

## Footnotes

^1^TD_5 is the sentence ID used to identify the sentence. The sentence ID is composed of text code_sequence number of the sentence in the text.

^2^In this article, square brackets are used to denote Spanish translations of Chinese expressions. These translations represent standard references rather than students’ attempts at sight translation.

^3^The asterisk* marks omitted information in parentheses.
